# Astragaloside IV Synergizing with Ferulic Acid Ameliorates Pulmonary Fibrosis by TGF-*β*1/Smad3 Signaling

**DOI:** 10.1155/2021/8845798

**Published:** 2021-03-02

**Authors:** Jiahuan Tong, Zhisong Wu, Yuchen Wang, Qingxun Hao, Haoge Liu, Fang Cao, Yang Jiao

**Affiliations:** ^1^Beijing University of Chinese Medicine, No. 11 Bei San Huan Dong Lu, Chaoyang District, Beijing 100029, China; ^2^Dongfang Hospital Affiliated to Beijing University of Chinese Medicine, No. 6 Fang Zhuang, Fengtai District, Beijing 100078, China

## Abstract

**Objective:**

The study aims to research the interventional effect and mechanism of astragaloside IV (Ast) synergizing with ferulic acid (FA) on idiopathic pulmonary fibrosis (IPF) induced by bleomycin in mice.

**Methods:**

The mice were randomly divided into seven groups with 10 mice in each group, namely, a sham operation group, a model group, a miRNA-29b (miR-29) group, a miR-29b negative control group (NC group), a FA group, an Ast group, and a combination group. A mouse model of pulmonary fibrosis was established by intratracheal instillation of bleomycin. Samples were collected after 28 days of continuous administration. Hematoxylin and eosin (HE) and Masson staining were used to observe pathological changes in the lung tissue, and the degree of fibrosis was evaluated using the hydroxyproline content. Changes in transforming growth factor-*β*1 (TGF-*β*1) and Smad3 in the lung were observed using immunohistochemistry. Enzyme-linked immunosorbent assay (ELISA) was used to detect the level of reactive oxygen species (ROS), malondialdehyde (MDA), and superoxide dismutase (SOD) in the serum. PCR was used to detect the expression of the miR-29b, TGF-*β*1, Smad3, and nuclear factor E2-related factor 2 (Nrf2) genes. Western blotting was used to detect the content of the TGF-*β*/Smad3 protein.

**Results:**

Ferulic acid combined with astragaloside IV reduced the degree of pulmonary fibrosis and the synthesis of hydroxyproline in lung tissue. The combination of the two also regulated the oxidative stress response ， TGF-*β*1/Smad3 pathway and miR-29b in lung tissue.

**Conclusion:**

Astragaloside IV combined with ferulic acid regulated the oxidative stress of lung tissues and TGF-*β*1/Smad3 signaling through miR-29b, thereby reducing the degree of pulmonary fibrosis. This provides a reference direction for the clinical treatment of IPF patients.

## 1. Introduction

Idiopathic pulmonary fibrosis (IPF) is a disease of unknown origin with progressive and diffuse pulmonary fibrosis accompanied by honeycomb cyst-like changes. The median survival time of patients is only two to three years, and the mortality rate is nearly the same as that of malignant tumors [1]. Currently, glucocorticoids and immunosuppressive agents are the primary treatment methods, but the treatment effect is not ideal and adverse reactions are obvious. Therefore, we need to conduct more research on the pathogenesis and treatment of IPF.

The pathogenesis of pulmonary fibrosis is not clear, but the role of miRNA in the pathogenesis of IPF has been paid an increasing amount of attention. Studies have shown that miR-29 is the most obvious miRNA molecule in IPF lung tissue [2]. MiR-29 can inhibit the synthesis of many components in the extracellular matrix and has an obvious antifibrotic effect [3]. MiR-29 in lung tissue is related to oxidative stress, the expression of the transforming growth factor-*β*1(TGF-*β*1)/Smad3 signal transduction pathway. TGF-*β*1 promotes the proliferation and transformation of fibroblasts. It can induce tissue fibrosis through endothelial mesenchymal transition [4]. The Smad3 protein is a type of signal protein that has been found to be involved in TGF-*β*1 signal transduction in recent years, and it is an important pathway of TGF-*β*1 signal transduction [5]. Oxidative stress induced by malondialdehyde (MDA) and superoxide dismutase (SOD) can activate TGF-*β*1/Smad3 signal transduction and participate in the occurrence of fibrosis. Oxidative stress and the TGF-*β*1/Smad3 signal interact with other processes and rapidly promote IPF. Therefore, TGF-*β*1/Smad3 may have a therapeutic effect on IPF.

The Danggui Buxue Decoction has a good antifibrosis effect, and it can reduce pulmonary fibrosis by improving the antioxidant system [6, 7]. Modern pharmacological studies have demonstrated [8] that astragaloside IV (Ast) and ferulic acid (FA) are the effective components of the Danggui Buxue Decoction that inhibit fibrosis. Astragaloside IV can improve the level of SOD, reduce the content of MDA, and activate Nrf2 antioxidant signaling pathway [9, 10]. Ferulic acid inhibits the secretion of activated TGF-*β* 1 [11, 12]. The cooperation of ferulic acid and astragaloside IV can inhibit fibrosis by regulating the nuclear factor E2-related factor 2 (Nrf2) and TGF-*β*1/Smad3 pathways [13]. They maintain a synergistic antifibrosis effect in rats with obstructive nephropathy [14]. There is evidence from clinical practice and experimental research that a Chinese herbal medicine mixture has an enhanced curative effect than a single drug, and the combination of astragaloside IV (Ast) and ferulic acid (FA) may also have a synergistic effect [15, 16]. The purpose of the study is to explore the therapeutic effects of astragaloside IV and ferulic acid on pulmonary fibrosis and their synergistic mechanism.

## 2. Materials and Methods

### 2.1. Chemicals

Astragaloside IV (purity ≥98%) and ferulic acid (purity≥98%) were bought from Shanghai yuan ye Bio-Technology Co., Ltd. (Shanghai, China). MIR-29 b agomir and agomir negative control were bought from Guangzhou Ribobio Co., Ltd. (Guangzhou, China). The assay kits of MDA, ROS, and SOD for mice were purchased from Jiancheng Bioengineering Institute (Nanjing, China). The HYP kit was bought from Nanjing Jiancheng Bioengineering Co., Ltd. (Nanjing, China).

### 2.2. Animals

C57BL male mice weighing 22 to 24 g were bought from Beijing Vital River Labomouseory Animal Technology Co., Ltd. (Beijing, China). The mice were  housed under laboratory conditions . The temperature was kept at 22–26°C, and the ambient humidity was kept at 50–60%. The food and water were free to mice. 

### 2.3. Animal Model and Experimental Design

A total of 70 mice were randomly divided into seven groups (*n* = 10 per group): a sham group, a model group, a NC group, a miR-29 group, a FA group, an Ast group, and a combination group.

The mice were anesthetized with isoflurane solution, and then bleomycin solution was instilled from the airway according to the standard of 2 u/kg, except for mice in the sham group, which received the same volume of saline solution instead.

On the second day after modeling, the mice in the miR-29 group and NC group were administered intratracheally with miR-29b agomir or miR-29b agomir negative control (10 mg/kg) every three days. Meanwhile, the mice in the FA group, Ast group, and combination group were, respectively, administered with ferulic acid (24 mg/kg), astragaloside IV (40.8 mg/kg), and the mixture of astragaloside IV and ferulic acid. The mice in the sham and model group were administered with the same amount of saline instead. The mice were going to be euthanized after 28 days.

### 2.4. Hematoxylin and Eosin (HE) Staining

Paraffin sections of the lung tissue specimens were routinely prepared. The paraffin sections were placed in a 30°C incubator for one hour and then deparaffinized using xylene and graded ethanol. Coat the paraffin sections on a glass slide and stain with hematoxylin for 5–10 minutes and eosin for 2–3 minutes. A high-resolution image analyzer is used for the histological analysis of specimens.

### 2.5. Masson's Stains

Masson staining is a method used to show collagen fibers and inflammatory factors in tissues. Paraffin sections of the lung tissues were routinely made and stained with Masson tricolor blue. The specimens were stained with hematoxylin for at least 6 min, dyed with the Masson compound oil for 5 min, and dyed with 5% phosphomolybdic acid for 5 min, and then 2% aniline blue was used for 10 min. The specimens were mounted on a slide after conventional dehydration.

### 2.6. Immunohistochemical Staining

Paraffin sections were routinely prepared. Then, slices were removed and placed in a drying oven at 37°C for 1 h. The antigen was recovered for 40 min, and then the endogenous peroxidase was quenched using a 3% H_2_ O_2_ solution for 10 min and kept away from light. The sections were incubated with the primary antibody for 20 min and then washed thoroughly with phosphate-buffered solution (PBS). They were then washed with the secondary antibody for 20 min and washed again. Positive immunostaining in the tissue was conducted using 3,3-diaminobenzidine tetrahydrochloride (DAB), and the staining of each section was observed.

### 2.7. Enzyme-Linked Immunosorbent Assay (ELISA)

ELISA was used to detect the level of ROS, MDA, and SOD in serum. The operation process is carried out in accordance with the instructions.

### 2.8. RT-PCR

The primers were designed as follows: actin 5-CTCCTGAGCGCAAGTACTCT-3 (forward) and 5-TACTCCTGCTTGCTGATCCAC-3 (reverse), TGF-*β*1 5-TTGCTTCAGCTCCACAGAGA-3 (forward) and 5-CAGAAGTTGGCATGGTAGCC-3 (reverse), Smad3 5-GGCTACCTTCCAGACCAACT-3 (forward) and 5-CCACTGCACTCCATAAGCAC-3 (reverse), Nrf2 5-TCCCAGCAGGACATGGATTT-3 (forward) and 5-GGCCTTCTCCTGTTCCTTCT-3 (reverse), miR-29b 5: GGGTAGCACCATTTGAAA-3 (forward) and 5- AACTGGTGTCGTGGAGTCGGC-3 (reverse).

### 2.9. Western Blot

The Western blot assay was used to analyze the expression levels of the TGF-*β*1, Smad3, P-Smad3, and Nrf2 proteins in the lung tissue. Fully lysed lung tissue samples with a RIPA lysis buffer were utilized. Then, the homogenate was centrifuged at 12000 r/min for ten minutes, and the supernatant was removed. The supernatant was boiled in an SDS sample buffer (MDL) for 5 min to denature the protein. Then, an equal amount of protein was separated on 10% SDS-PAGE (Anngen, catalog number Da2604) and transferred to polyvinylidene fluoride (PVDF). The membrane was blocked with 5% skimmed milk for 2 h and then incubated with primary antibodies (TGF-*β*1, Smad3, P-Smad3, and Nrf2) at 4°C overnight. After 60 min of exposure to the secondary antibody at 37°C, the membrane was analyzed using an ECL reagent. All of the determinations were performed independently and repeated three times.

### 2.10. Statistical Analysis

SPSS 21.0 was used for the statistical analyses. A one-way analysis of variance (ANOVA) was used to analyze the data between different groups. The least significant difference (LSD) method was used for the mean square error, and Dunnett T3 was used for the square difference quality. *p* < 0.05 represents statistical significance.

## 3. Results

### 3.1. Deaths and Body Weight Changes

**Figure 1:**
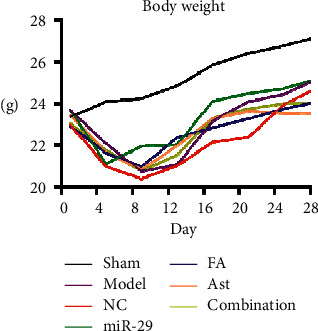
Changes in body weight of mice.

No mice died in the sham group. A mouse died on days 11 and 16 in the model group. A mouse died on days 8, 13, 20, and 26, in the NC group. In the miR-29 group, one mouse died on day 14. In the FA group, one mouse died on day 10. A mouse died on day 21 in the Ast group. In the combination group, one mouse died on day 20. The numbers of deaths in the model group, NC group, miR-29 group, FA group, Ast group, and the combination group were 2, 3, 2, 1, 1, and 1, respectively (Figure 1).

**Figure 2:**
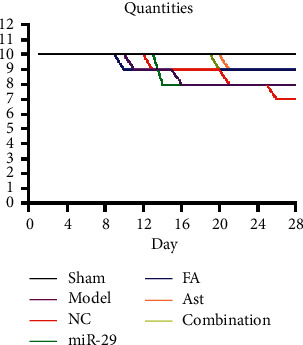
Changes in quantities of mice.

In the sham group, the body weight of the mice increased. On the fifth day, statistical differences appeared between the sham group and the NC group (*p* < 0.05), the sham group and the miR-29 group (*p* < 0.05), and the sham group and the FA group (*p* < 0.05). On the ninth day, statistical differences appeared between the sham group and the model group (*p* < 0.05) and the sham group and the NC group (*p* < 0.05). On the 13^th^ day, a statistical difference appeared between the sham group and the NC group (*p* < 0.05) , the Ast groupand the model group (P < 0.05), the Ast group and NC group (P < 0.05). (Table 1, Figure 2).

### 3.2. HE Staining and Masson's Staining

**Figure 3:**
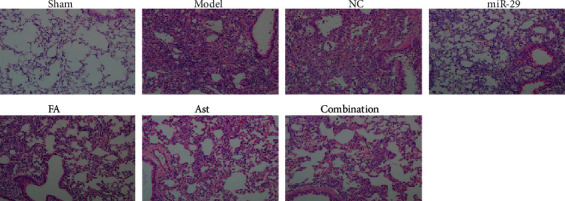
Changes in the lung of mice by HE staining (×200).

**Figure 4:**
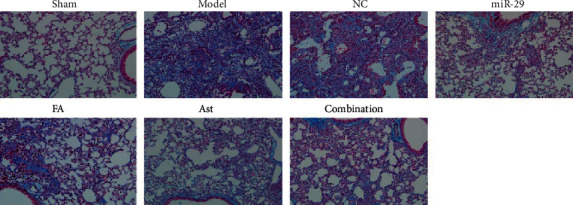
Changes in the lung of mice by Masson staining (×200).

In the lungs of the sham group, the histopathological staining results showed normal lung tissue, no inflammatory exudation, and no fibrous deposition. In the model and NC groups, the lungs were seriously injured with inﬂammatory cell infiltration, extensive collagen ﬁber deposition, and thickening of the pulmonary interstitium present. The treatment groups reduced inflammatory exudation and collagen fiber deposition in lung tissues (Figures 3 and 4).

### 3.3. Immunohistochemical Staining

Compared with the sham group, the expressions of TGF-*β*1 in the model group and the NC group were increased and were primarily concentrated in the nucleus. This suggested that the content of TGF-*β*1 increased during pulmonary fibrosis. The contents of TGF-*β*1 in the treatment groups were reduced to a certain extent.

**Figure 5:**
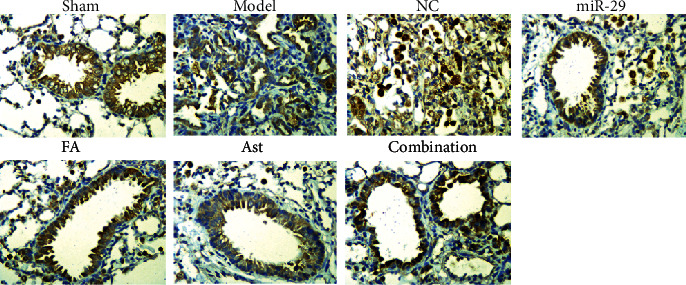
Expression of TGF-*β*1 in lung tissue *via* immunohistochemistry (IHC × 400).

**Figure 6:**
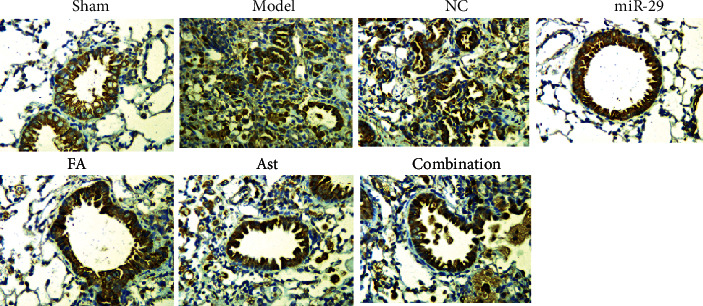
Expression of smad3 in lung tissue *via* immunohistochemistry (IHC × 400).

Compared with the sham group, the expressions of smad3 in the model group and the NC group were significantly increased and primarily concentrated in the cytoplasm. This result demonstrated that, under the promotion of TGF-*β*1, more smad3 was synthesized and released. The smad3 contents in the lung tissues of the treatment groups were significantly reduced (Figures 5 and 6).

### 3.4. The Effect of Ferulic Acid and Astragaloside IV on the Amount of Hydroxyproline

**Figure 7:**
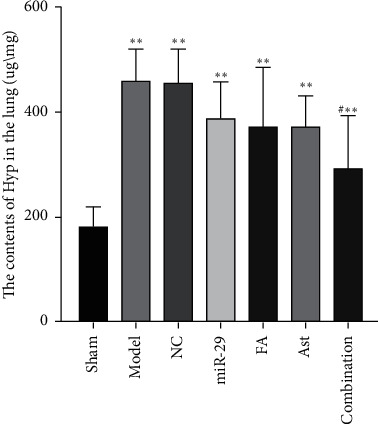
The content of Hyp in the lung. ^*∗∗*^*p* < 0.01 vs. Sham; ^#^*p* < 0.05 vs. model.

The amount of hydroxyproline (Hyp) in the lung tissues was measured on day 28. Compared with the sham group, the contents of Hyp of the lung tissues in the model, NC, miR-29, FA, Ast, and combination groups increased (*p* < 0.01, vs. Sham). Compared with the model group, the combination group had a significantly decreased content of Hyp (*p* < 0.05, vs. the model) (Figure 7).

### 3.5. The Effect of Astragaloside IV and Ferulic Acid on the SOD, ROS, and MDA Levels in the Serum

**Figure 8:**
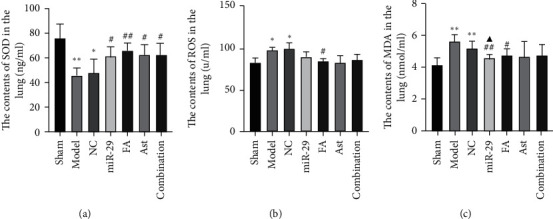
The serum levels of SOD, ROS, and MDA. (a) The level of SOD. (b) The level of ROS. (c) The level of MDA. ^*∗*^*p* < 0.05 versus Sham; ^*∗∗*^*p* < 0.01 versus Sham; ^#^*p* < 0.05 versus model; and ^Δ^*p* < 0.05 versus  NC.

Compared with the sham group, the content of SOD, ROS, and MDA in the model and NC groups had statistical differences (*p* < 0.05 or 0.01 vs. the sham). Compared with the model group, the levels of SOD in the miR-29, FA, Ast, and combination groups were notably increased (*p* < 0.05 or 0.01 vs. the model), the level of ROS in the FA group decreased (*p* < 0.05 vs. the model), and the levels of MDA in the miR-29 and FA groups were remarkably decreased (*p* < 0.05 or 0.01 vs. the model). Compared with the NC group, the level of MDA in the miR-29 group decreased (*p* < 0.05 vs. the NC) (Figure 8).

### 3.6. The Effect of Ferulic Acid and Astragaloside IV on the mRNA Levels of TGF-*β*1, Smad3, Nrf2, and miR-29b in the Lung

**Figure 9:**
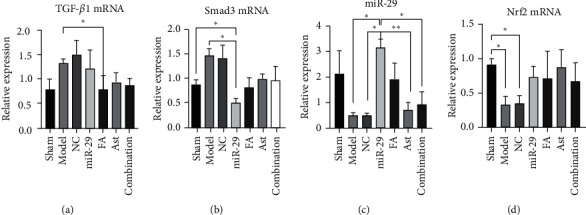
The mRNA levels of TGF-*β*1, Smad3, Nrf2, and miR-29b. (a) The level of TGF-*β*1. (b) The level of Smad3. (c) The level of Nrf2. (d) The level of miR-29b. ^*∗*^*p* < 0.05; ^*∗∗*^*p* < 0.01.

Compared with the model group, the level of TGF-*β*1 mRNA in the FA group decreased significantly (*p* < 0.05 vs. the model). The miR-29 group showed an apparent decrease in the level of Smad3 mRNA (*p* < 0.05 vs. the sham and model). The miR-29 group displayed a significant increase in the level of miR-29b as compared with the model, NC, Ast, and combination groups (*p* < 0.05 or 0.01). In the model group and the NC group, the level of Nrf2 mRNA decreased remarkably (*p* < 0.05 vs the sham group). (Figure 9).

### 3.7. The Effect of Astragaloside IV and Ferulic Acid on the Protein Levels of TGF-*β*1, Smad3, p-Smad3, and Nrf2 in the Lung Tissues

**Figure 10:**
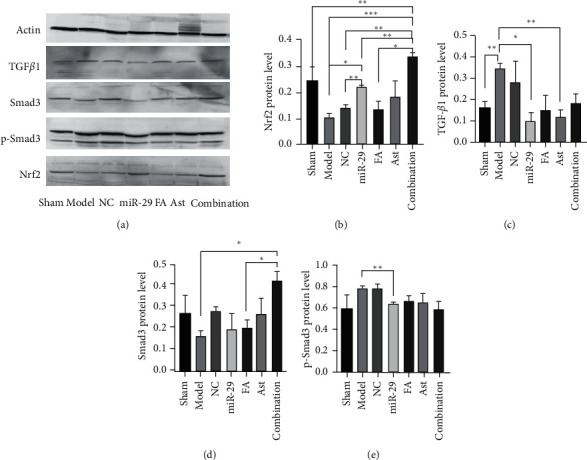
The protein levels of TGF-*β*1, Smad3, p-Smad3, and Nrf2. ^*∗*^*p* < 0.05; ^*∗∗*^*p* < 0.01.

Compared with the sham, model, NC, miR-29, and FA groups, the levels of Nrf2 expression in the combination group increased remarkably (*p* < 0.05 or 0.01). Compared with the model and the NC groups, the level of Nrf2 protein in the miR-29 group remarkably increased (*p* < 0.05 or 0.01). Compared with the sham group, the level of TGF-*β*1 protein in the model group increased significantly (*p* < 0.01). Compared with the model group, the level of TGF-*β*1 expression in the miR-29 group and Ast group decreased (*p* < 0.05 or 0.01). In the combination group, the level of Smad3 expression increased significantly, compared with the model and FA groups (*p* < 0.05). Compared with the model group, the level of p-Smad3 expression in the miR-29 group decreased with a statistical difference (*p* < 0.01) (Figure 10).

## 4. Discussion

The pathogenesis of pulmonary fibrosis is very complicated. Existing studies believe that a variety of factors take significant parts in the occurrence and development of pulmonary fibrosis. MicroRNA is a kind of noncoding single-stranded small RNA that regulates protein expression after transcription through complementary pairing with mRNA. Studies have found that the expression of multiple mRNAs can be regulated by the same type of microRNA, and the same mRNA may also be regulated by different microRNAs [17, 18]. The family of miR-29 consists of three members: miR-29a, miR-29b, and miR-29c. There are only two to three nucleotide differences between these three members, and they have the same seed sequence. The target genes of action are often the same and are highly conservative [19]. MiR-29 can directly inhibit the production of collagen fibers or inhibit pulmonary fibrosis through a variety of cytokines [20]. Oxidative stress is an important mechanism that triggers the formation of pulmonary fibrosis. After lung injury, quantities of reactive oxygen species (ROS) are released, which cause peroxidation of the lung tissue and stimulate the synthesis and secretion of collagen [21]. However, ROS can also increase the level of connective tissue growth factor and matrix metalloproteinases by regulating the TGF-*β*1/Smad3 signaling pathway and promoting extracellular matrix remodeling and pulmonary fibrosis [22, 23]. Studies have shown that the overexpression of the miR29 family can reduce the contents of ROS and MDA, eliminate oxidative stress products, significantly increase the content of superoxide dismutase (SOD), and increase the body's antioxidant levels, thereby reducing the degree of pulmonary fibrosis. MiR-29 can also directly act on the 3′-UTR of the target gene TGF-*β*1 [24], inhibit the transcription of TGF-*β*1 mRNA, and reduce pulmonary fibrosis through the inhibitory effect of the TGF-*β*1/Smad3 signaling pathway [5].

The bleomycin animal model used in this experiment has been widely used to study IPF and has been recognized by many scholars [25]. The mice models in this study were induced using endotracheal bleomycin administration, and then obvious pulmonary fibrosis was shown. In this experiment, the characteristics of IPF in mice are consistent with other studies [26, 27].

After 28 days of administration, the lung histopathologies of each treatment group improved. The HE and Masson staining showed that a large number of inflammatory exudates were seen in the lung tissues of the model group and the NC group, with a widened alveolar septum, congestion, severe alveolar destruction, a large number of collagen fibers and deposition, and even the formation of fibrous nodules. In the treatment group, the degree of inflammatory infiltration was lighter, with a small amount of collagen fiber deposited. In addition, the degree of pulmonary fibrosis was significantly reduced. Therefore, it can be speculated that ferulic acid and astragaloside IV had an effect on improving pulmonary fibrosis.

HYP, as an important biomarker of collagen, has an important impact on the deposition and remodeling of extracellular matrix (ECM). Compared with the model group, the HYP in the lung tissues of each treatment group decreased, especially in the combination group. This experimental study showed that, compared with the model group, both astragaloside IV and ferulic acid reduced the content of HYP to a certain extent. And the effect of the combination was better.

ELISA results showed that, compared with the model group, astragaloside IV, ferulic acid, and the combination of the two significantly increased the level of SOD, and ferulic acid also significantly reduced the level of ROS and MDA. This suggested that astragaloside IV and ferulic acid regulated the oxidation reaction of pulmonary fibrosis, and increased the level of antioxidant factors SOD, and ferulic acid reduced the levels of oxidation factors ROS and MDA.

The results of the RT-PCR showed the contents of mRNA of the TGF-*β*1, smad3, miR-29b, and Nrf2. In the model group, the contents of TGF-*β*1 and Smad3 increased and the contents of miR-29b and Nrf2 decreased, compared with the sham group. Compared with the model group, the level of TGF-*β*1 mRNA decreased significantly, and the miR-29b increased in the FA group. At the same time, the modulatory effect of the combination group was moderate. These results indicated that ferulic acid and astragaloside IV regulated the expression of TGF-*β*1, Smad3, miR-29b, and Nrf2 at the gene level, and the combination of FA and Ast had a certain synergistic effect.

Western blotting was used to detect the protein content of TGF-*β*1, Smad3, p-Smad3, and Nrf2 in the lungs. Compared with the sham group, TGF-*β*1 and p-Smad3 of the model group increased to a certain extent, and the content of Nrf2 decreased. Compared with the model group, the content of TGF-*β*1 of the Ast group was significantly reduced, and the FA and combination group also had a certain degree of reduction. The result showed astragaloside IV and ferulic acid can reduce TGF-*β*1 protein content and reduce pulmonary fibrosis. Although the content of smad3 in the combination group was increased, the content of p-Smad3 was lower than that in the model group. This result suggested that astragaloside IV and ferulic acid may have interfered with the phosphorylation pathway of Smad3 and adjusted the level of p-Smad3 to interfere with the process of pulmonary fibrosis. Immunohistochemistry also suggested that the synthesis of TGF-*β*1 and smad3 increased in the model group, and most of them gathered in the nucleus and cytoplasm. Both astragaloside IV and ferulic acid reduced their contents to a certain extent. On the other hand, the level of Nrf2 in the model group was lower than that in the sham group, while the level of Nrf2 in the treatment groups was higher than that in the model group. The growth of the combination group was the most obvious, even surpassing the miR-29 group. This suggested that astragaloside IV and ferulic acid can interfere with the oxidative reaction process involved in Nrf2 and reduce lung damage, and they have a synergistic effect.

## 5. Conclusions

The combination of astragaloside IV and ferulic acid reduced oxidative stress, collagen synthesis, and inhibited the TGF-*β*1/Smad3 signaling pathway through miR-29b regulation, thereby reducing the damage from pulmonary fibrosis. This study provides an experimental basis for the combination of ferulic acid and astragaloside IV in the treatment of pulmonaryfibrosis. However, more investigationsare required to study the underlying mechanisms in detail.

## Figures and Tables

**Table 1:** Comparison of body weight changes in 7 groups. ^*∗*^*p* < 0.05 versus Sham; ^#^*p* < 0.05 versus  model; ^Δ^*p* < 0.05 versus  NC.

Group	1^st^ day	5^th^ day	9^th^ day	13^th^ day	17^th^ day	21^st^ day	25^th^ day	28^th^ day
Sham	23.35 ± 1.31	24.09 ± 1.49	24.24 ± 1.47	24.85 ± 1.31	25.86 ± 1.4	26.42 ± 1.37	26.77 ± 1.37	27.11 ± 1.39
Model	23.68 ± 1.5	22.08 ± 1.89	20.74 ± 2.1^*∗*^	21.07 ± 2.87	23.15 ± 1.94	24.09 ± 2.14	24.43 ± 2	25.04 ± 2.03
NC	22.98 ± 1.33	20.99 ± 2.2^*∗*^	20.38 ± 2.87^*∗*^	21.02 ± 3.41^*∗*^	22.14 ± 3.95	22.39 ± 4.45	23.94 ± 2.49	24.59 ± 2.18
miR-29	23.7 ± 1.07	21.11 ± 3.28^*∗*^	21.95 ± 2.07	22.02 ± 4.43	24.12 ± 2.11	24.47 ± 1.86	24.72 ± 1.96	25.1 ± 1.83
FA	22.93 ± 2.05	21.63 ± 1.67^*∗*^	20.97 ± 1.82	22.36 ± 1.92	22.85 ± 1.83	23.27 ± 1.89	23.74 ± 1.59	24.02 ± 1.35
Ast	23.46 ± 1.36	21.66 ± 1.72	20.83 ± 2.3	21.97 ± 2.63^▲#^	23.28 ± 1.93	23.62 ± 1.8	23.54 ± 1.33	23.51 ± 1.64
Combination	23.06 ± 1.22	21.76 ± 1.77	20.76 ± 2^*∗*^	21.53 ± 2.9	23.3 ± 1.53	23.74 ± 1.71	24 ± 1.63	24.02 ± 2.36

## Data Availability

The data used to support the findings of this study are included within the article.
